# A Veiled Lymphatic Malformation: Stridor in a Child

**DOI:** 10.7759/cureus.67461

**Published:** 2024-08-22

**Authors:** Rathakrishnan Venkatasamy, Bee See Goh, Rufinah Teo, C-Khai Loh

**Affiliations:** 1 Department of Otorhinolaryngology - Head and Neck Surgery, Faculty of Medicine, Universiti Kebangsaan Malaysia, Kuala Lumpur, MYS; 2 Department of Otorhinolaryngology - Head and Neck Surgery, Hospital Canselor Tuanku Muhriz UKM, Kuala Lumpur, MYS; 3 Department of Anaesthesiology and Intensive Care, Faculty of Medicine, Universiti Kebangsaan Malaysia, Kuala Lumpur, MYS; 4 Department of Paediatrics, Faculty of Medicine, Universiti Kebangsaan Malaysia, Kuala Lumpur, MYS

**Keywords:** head and neck, pediatric, stridor, neck mass, lymphatic malformation

## Abstract

Lymphatic malformation (LM) is a congenital lymphatic dysplasia associated with the p110α subunit of PI3K (PIK3CA) mutation. A two-year-old boy presented with a history of noisy breathing from the age of two months, which was progressively worsening. Inspiratory stridor was audible with subcostal recession. Flexible nasopharyngolaryngoscopy (FNPLS) revealed an enlarged right arytenoid. Other supraglottic structures were normal, and bilateral vocal cords were mobile. Direct laryngoscopy showed that the right arytenoid was enlarged with a smooth surface. On the subsequent visit, there was a painless soft lateral neck swelling, 4 cm x 4 cm in size, with normal skin. MRI confirmed LM with the predominantly macro-cystic component, involving primarily the right neck and upper mediastinum, causing airway compression. Sirolimus therapy was initiated, and at one month of follow-up after the treatment, his stridor had improved. The incidence of stridor secondary to head and neck tumors such as teratomas, hemangiomas, and LM accounts for less than 3%. The typical manifestation of LM often involves a painless, soft, and compressible mass that progressively increases in size. Features of macrocystic LM on MRI are multilocular and hyperintense cystic mass on T2-weighted imaging. The treatment methods for LM include surgical and non-surgical options. Despite being an off-label application, the response rate of sirolimus therapy in children with LM is reported to be 91%, and the first clinical response was observed in less than three weeks. Stridor is frequently encountered in children but rarely due to head and neck tumors. However, as in our case, a large LM may cause recurrent airway obstruction, and the neck swelling may appear later. Atypical airway findings, especially endoscopic examination, in a child with stridor should be complemented with imaging to examine the possibility of extra-laryngeal mass or external compression.

## Introduction

Lymphatic malformation (LM) is one of the simple vascular malformations based on the International Society for the Study of Vascular Anomalies (ISSVA) classification. It is a congenital lymphatic dysplasia associated with PIK3CA mutations [1,2]. The estimated prevalence is one in every 4,000 live births [3]. LM more than 1 cm is categorized as macrocystic, smaller lesions are termed microcystic, and a combination of both may be present in the same patient [4]. Most LMs occur in the head and neck region due to its rich lymphatic supply. Most LMs are present at birth, and 90% are diagnosed before the age of three. The commonest manifestation of LM is a painless soft mass gradually enlarging in size. A large LM may cause stridor but rarely manifests alone [5,6]. Imaging such as ultrasound, computed tomography (CT), and magnetic resonance imaging (MRI) are vital in diagnosing LM [7]. This case report highlights the unique presentation, physical examination, imaging studies, and management of an infant diagnosed with LM in the oropharynx, neck, and mediastinum.

## Case presentation

We present a case of head and neck LM in a two-year-old boy. He presented with a history of noisy breathing from the age of two months, which was progressively worsening. The noise had been persistent and louder during exertion. It was not associated with rapid breathing or cyanotic spells, but parents have noticed intermittent chest recession. His cry had been perceived as normal by his parents. In terms of feeding, there was no interruption or choking, and his weight gain has been appropriate for his age. In the past, he had been admitted thrice to the hospital due to pneumonia at the age of nine months, 10 months, and 18 months before referral to our center. He received non-invasive ventilation support for the respiratory distress that developed during the admissions but no history of intubation. Antenatal history was unremarkable. He was born via emergency Caesarean section for fetal distress at term. The delivery was uneventful, and no neck mass was identified upon delivery.

**Figure 1:**
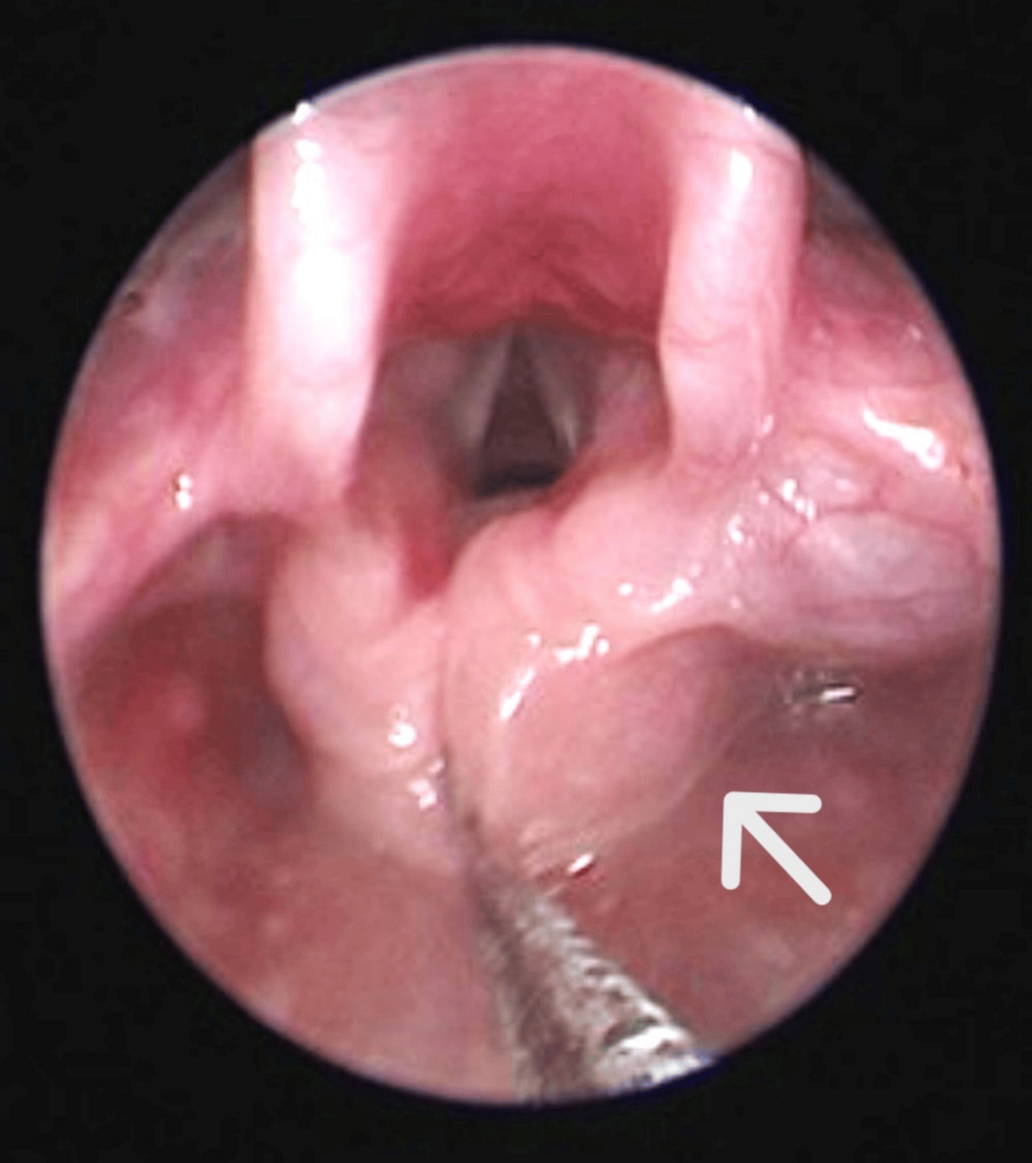
The right arytenoid (arrow) is enlarged and displaced anteriorly.

Upon physical assessment, the inspiratory stridor was audible with subcostal recession. There were no craniofacial anomalies or cutaneous hemangioma observed. The neck, oral cavity, and lung examinations were unremarkable. Flexible nasopharyngolaryngoscopy (FNPLS) performed at our clinic revealed an enlarged right arytenoid compared to the left arytenoid. Other supraglottic structures were normal and bilateral vocal cords were mobile. Subsequently, direct laryngoscopy and bronchoscopy were performed under general anesthesia. Intra-operative assessment of the larynx showed that the right arytenoid (Figure 1) was enlarged and displaced anteriorly with a smooth surface but difficult to assess the border as the swelling was seen extending into the paraglottic space. This raised the suspicion of an external mass possibly extending to the neck. Therefore, a magnetic resonance imaging (MRI) appointment was requested.

**Figure 2:**
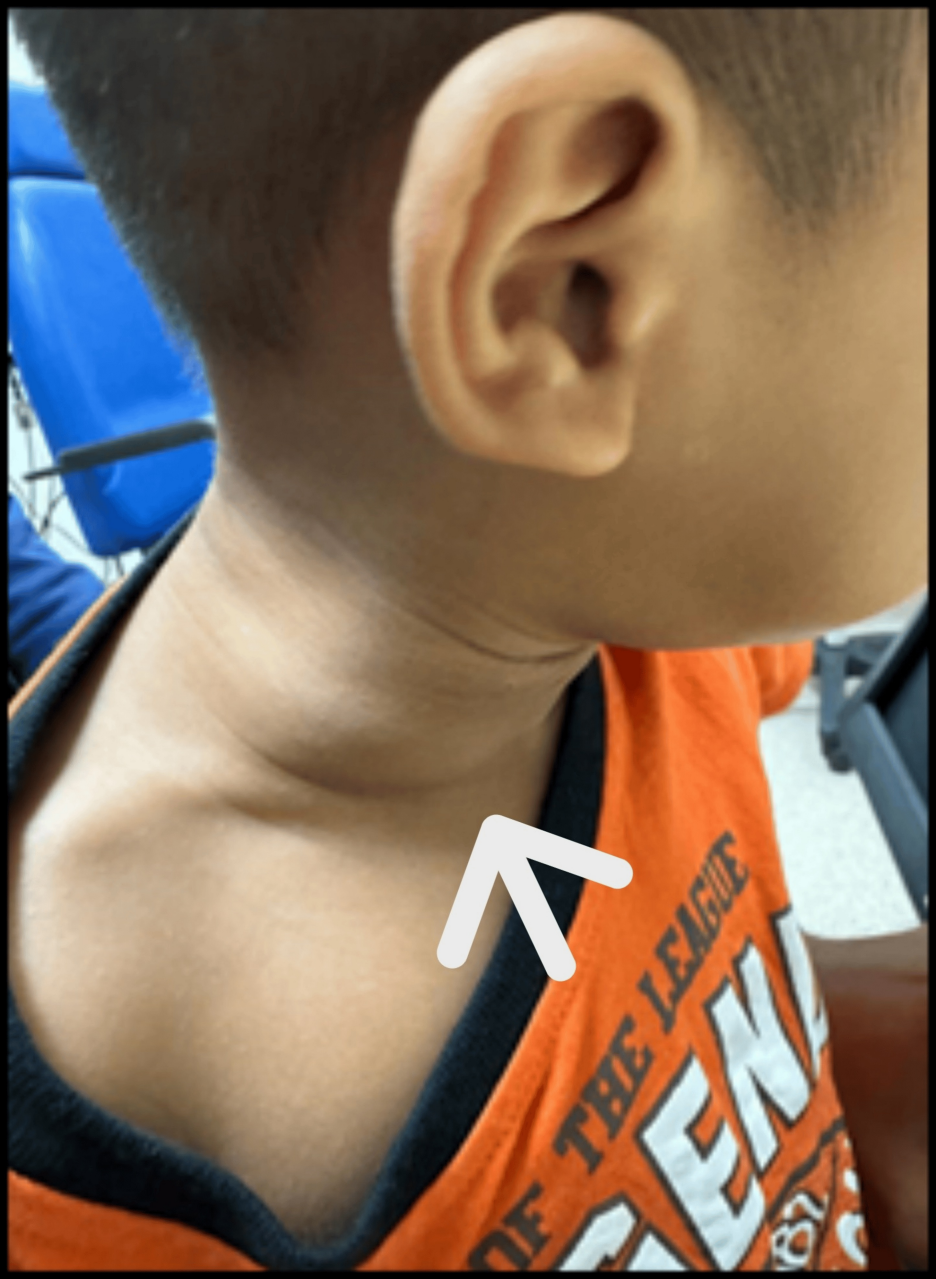
A lateral neck swelling (arrow), 4 cm x 4 cm in size, with normal overlying skin.

**Figure 3:**
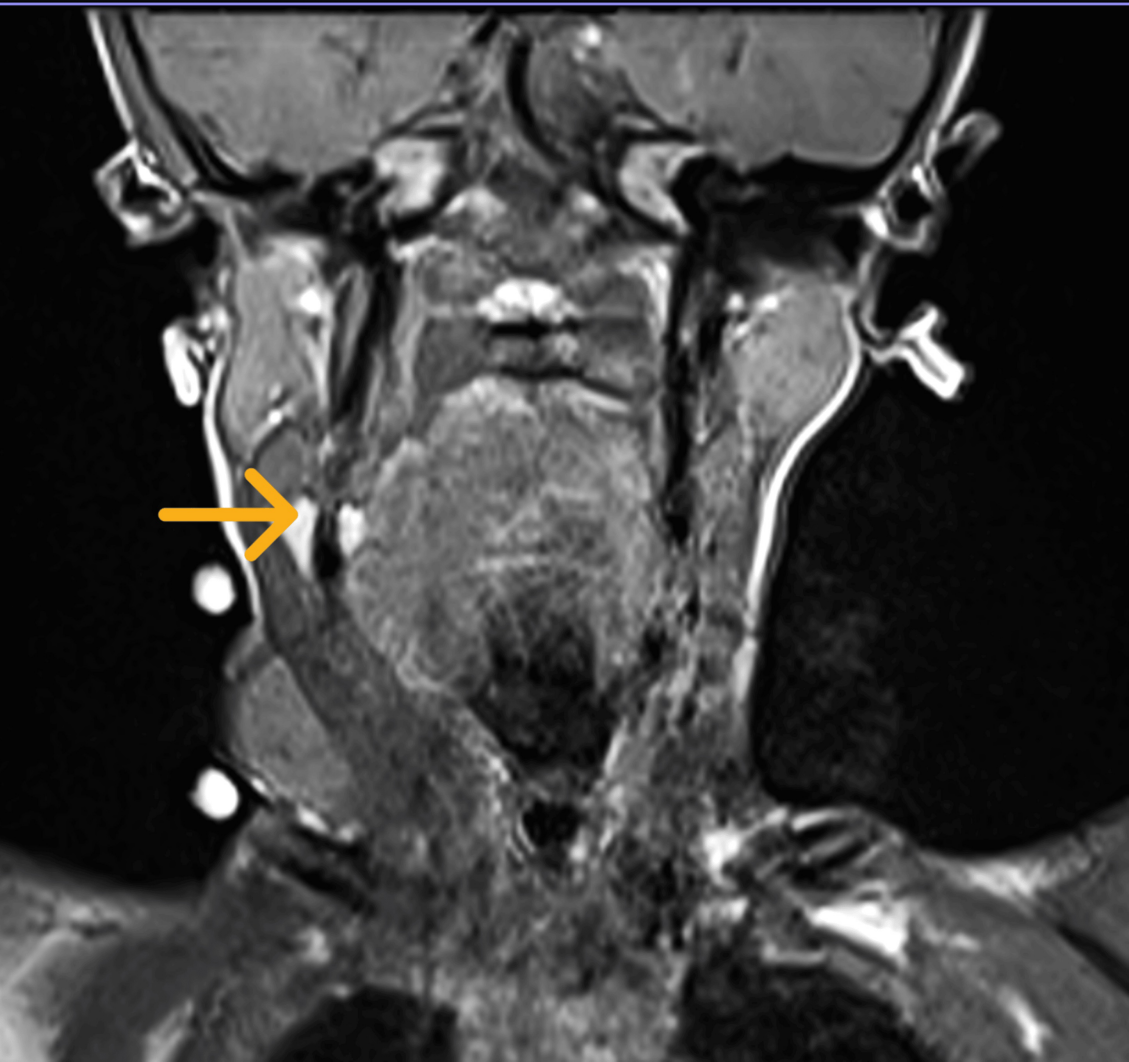
A coronal image of the MRI neck T1-weighted image with a hypointense signal (arrow).

**Figure 4:**
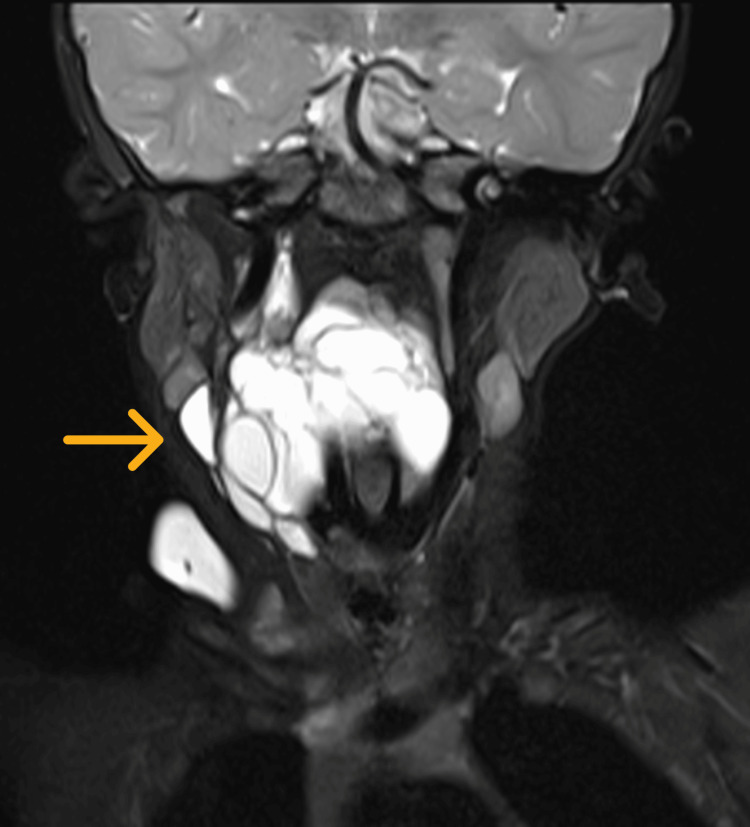
The same multiloculated cystic lesion shows a hyperintense signal (arrow) on an MRI neck T2-weighted image.

**Figure 5:**
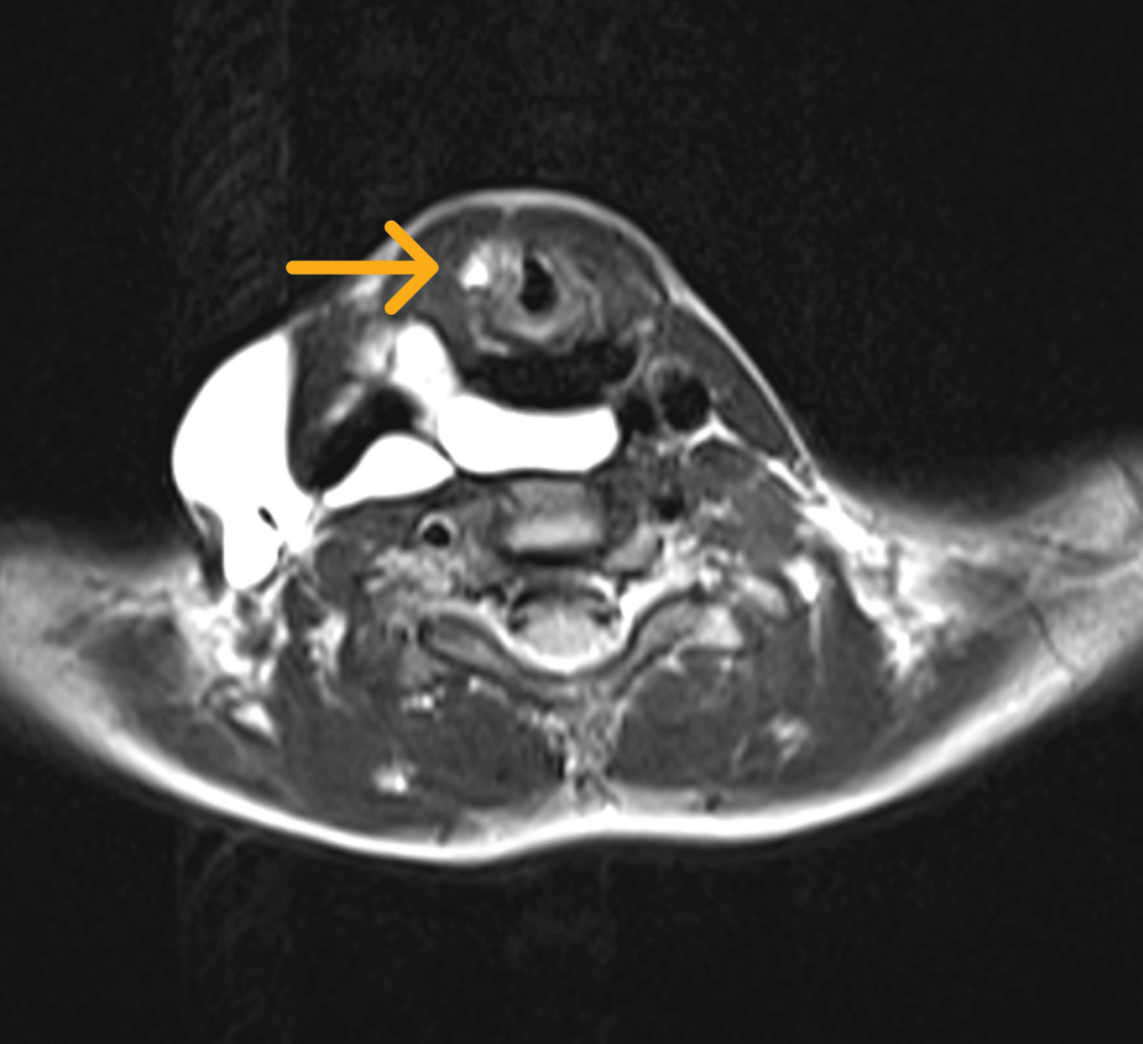
An axial view of the MRI neck shows an enhancing cystic lesion in the retropharyngeal region insinuating into the right carotid space and encircling the right sternocleidomastoid. The right aryepiglottic fold shows a hyperintense signal (arrow) and is displaced medially.

On the subsequent visit before the MRI appointment, parents expressed concern over a painless, progressively enlarging right neck swelling (Figure 2) prominent in distress. The lateral neck swelling was 4 cm x 4 cm in size, gradually enlarging, soft in nature, not tender, and no skin abnormalities. MRI unveiled a multiloculated cystic lesion measuring 2.4 cm x 5.1 cm x 9.7 cm extending from retropharyngeal space at the level of first to third thoracic vertebrae and insinuating into multiple fascial planes, predominantly to the right cervical and supraclavicular region. The lesion is hypointense on the T1-weighted image (Figure 3) and hyperintense on the T2-weighted image (Figure 4). In addition, rim enhancement is seen in the gadolinium study. Laterally, this lesion is causing splaying of the right common carotid artery and internal jugular vein. Medially, the retropharyngeal component is compressing the oropharynx and laryngopharynx, causing luminal narrowing and crossing midline into the left parapharyngeal space. Anteriorly, the right aryepiglottic fold (Figure 5) and supraglottic region show a hyperintense signal on the T2-weighted image. Pyriform fossae appear dilated with the presence of fluid. Inferiorly, the right supraclavicular cystic lesion is seen encompassing the right sternocleidomastoid muscle and right internal jugular vein. In summary, MRI is suggestive of LM with predominantly macro-cystic component, involving primarily the right neck and upper mediastinum causing airway compression.

After a multidisciplinary team discussion, a joint decision with parents was made to treat the child with sirolimus. Sclerotherapy was not preferred due to the potential inflammation surrounding the airway after injection, which may necessitate tracheostomy. Treatment was initiated, and at one month of follow-up post treatment, his parents said his stridor had improved. Clinically, there was no audible stridor at rest during the recent clinical visit. Sirolimus will be continued for a minimum duration of six or 12 months due to the extensiveness of the lesion. Therapeutic drug monitoring for sirolimus, full blood count, renal profile, liver function test, and lipid profile are routinely examined during monthly visits. Subsequently, surveillance will incorporate regular clinic visits, MRIs, and regular review of the child by the pediatrician for adverse reaction monitoring.

## Discussion

LMs are congenital abnormalities that begin to develop during prenatal development, typically in the sixth week of gestation. These malformations occur due to either the sequestration of embryonic lymphatic channels or inadequate drainage of the lymphatic sac into the jugular vein, resulting in congenital obstruction of lymphatic drainage [8]. Besides the atypical expression of molecules associated with lymph angiogenesis, including vascular endothelial growth factor (VEGF)-C and VEGF receptor type 3 (VEGFR3), recent advances in molecular biology have detected PIK3CA gene mutations in the lymphatic endothelial cells within the lesions [2].

Stridor is common in the pediatric group; common differential diagnoses are laryngomalacia, subglottic stenosis, vocal cord paralysis, and vallecular cysts [9,10]. The incidence of stridor secondary to head and neck tumors such as teratomas, hemangiomas, and LM accounts for less than 3% [10]. Large congenital head and neck tumors can be detected via antenatal ultrasound scan from the early second trimester and anticipate ex-utero intrapartum treatment mode of delivery [5]. In our case, the antenatal ultrasound scans were reported normal, and no neck mass was present upon birth. However, the child developed a neck swelling around the age of two. The typical manifestation of LM often involves a painless, soft, and compressible mass that progressively increases in size. The examination of the larynx did not show typical findings of common causes of stridor. In these circumstances, imaging is paramount to evaluating stridor, especially secondary to the external tumor.

Ultrasound is helpful to identify and assess cystic structures, especially macrocystic malformations. Ultrasound with Doppler is useful in detecting blood flow within a cyst. The absence of Doppler flow within a cyst distinguishes LM from hemangioma [6,7]. However, MRI can provide a more detailed view of the anatomical extent of a large macrocystic LM when compared to ultrasound. Features of macrocystic LM on MRI are a multilocular and hyperintense cystic mass on T2-weighted imaging and post-contrast enhancement within the locule. Microcystic LM shows hyperintense signals on T2-weighted imaging too, but no cystic spaces are visualized [6,7]. The imaging study of our patient displayed typical features of LM. Abscess may manifest with similar enhancement but can be ruled out by physical examination.

Contrary to hemangiomas, LM remains present throughout an individual’s life, increases in proportion to the patient’s size, and does not usually experience the natural regression observed in hemangiomas. Spontaneous regression of LM is reported to be lower than 15% [5,6]. Additionally, LM has a risk of infection and may result in acute swelling with respiratory distress. Disfigurement and social stigma are matters of concern too [11]. Therefore, monitoring alone may not be ideal for LM. Management of LM is undertaken by a multidisciplinary team. The treatment methods for LM include surgery and non-surgical options, such as pharmacotherapy, sclerotherapy, laser therapy, radiotherapy, electrocoagulation, cryotherapy, ligation, and embolization [5,12]. Surgery is optimum for localized microcystic lesions, but complete resection of extensive lesions is challenging. Sclerotherapy is advocated for simple macrocystic LM. It shows a response rate of more than 80% and complete involution in 40% of the cases [11]. The advantage of sclerotherapy is that it is repeatable, but the effect is seen after a few weeks [5]. Although the success of sclerotherapy is widely reported, the risk of inflammation post injection and the ensuing swelling are some concerns, especially for lesions surrounding the airway [7]. Despite being an off-label application, sirolimus has been utilized in treating vascular anomalies. Sirolimus treatment had a response rate of 91% in children with LM, and the first clinical response was observed in less than three weeks. Adverse effects of sirolimus are sporadically reported. A systematic review of 19 studies reported hyperlipidemia, neutropenia, and infections such as upper respiratory tract infection, cellulitis, and mucositis as common side effects [13]. The serum level of sirolimus can be tested, but the therapeutic and toxic levels remain unclear [4]. The commonest dosage administered was 0.8 mg/m² every 12 hours via the oral route, intending to attain a therapeutic serum level of 5-15 ng/mL [4,13]. There is a lack of standardization in the therapeutic blood concentration of sirolimus. Therefore, meticulous risk versus benefit analysis is necessary if opting for sirolimus therapy.

## Conclusions

Stridor is often seen in children but is rarely caused by head and neck tumors. However, as in our case, a large LM may lead to repeated airway blockage, and neck swelling may develop later. Unusual airway findings, particularly those discovered during endoscopic examination in a child with stridor, should be followed up with imaging to investigate the potential presence of masses outside the larynx or external compression.
